# Employment of Artificial Intelligence Based on Routine Laboratory Results for the Early Diagnosis of Multiple Myeloma

**DOI:** 10.3389/fonc.2021.608191

**Published:** 2021-03-29

**Authors:** Wei Yan, Hua Shi, Tao He, Jian Chen, Chen Wang, Aijun Liao, Wei Yang, Huihan Wang

**Affiliations:** ^1^ Haematology Department of Shengjing Hospital, China Medical University, Shenyang, China; ^2^ Neusoft Research Institute, Northeastern University, Shenyang, China

**Keywords:** multiple myeloma, artificial intelligence, early diagnosis, gradient boosting decision tree, machine learning

## Abstract

**Objective:**

In order to enhance the detection rate of multiple myeloma and execute an early and more precise disease management, an artificial intelligence assistant diagnosis system is developed.

**Methods:**

4,187 routine blood and biochemical examination records were collected from Shengjing Hospital affiliated to China Medical University from January 2010 to January 2020, which include 1,741 records of multiple myeloma (MM) and 2,446 records of non-myeloma (infectious diseases, rheumatic immune system diseases, hepatic diseases and renal diseases). The data set was split into training and test subsets with the ratio of 4:1 while connecting hemoglobin, serum creatinine, serum calcium, immunoglobulin (A, G and M), albumin, total protein, and the ratio of albumin to globulin data. An early assistant diagnostic model of MM was established by Gradient Boosting Decision Tree (GBDT), Support Vector Machine (SVM), Deep Neural Networks (DNN), and Random Forest (RF). Out team calculated the precision and recall of the system. The performance of the diagnostic model was evaluated by using the receiver operating characteristic (ROC) curve.

**Results:**

By designing the features properly, the typical machine learning algorithms SVM, DNN, RF and GBDT all performed well. GBDT had the highest precision (92.9%), recall (90.0%) and F1 score (0.915) for the myeloma group. The maximized area under the ROC (AUROC) was calculated, and the results of GBDT (AUC: 0.975; 95% confidence interval (CI): 0.963–0.986) outperformed that of SVM, DNN and RF.

**Conclusion:**

The model established by artificial intelligence derived from routine laboratory results can accurately diagnose MM, which can boost the rate of early diagnosis.

## Introduction

As a hematological malignancy, multiple myeloma (MM) accounts for 1% of all cancer and 13% of hematological tumors with the characteristics of proliferation of malignant plasma cells in the bone marrow (BM), presence of anemia, renal dysfunction, hypercalcemia, and lytic lesions (1). The involvement of other disciplines such as orthopedics, nephrology and hematology often cause misdiagnoses (2). In addition, due to the sub-par distribution of state-of-the-art medical and diagnostic equipment, rural health centers and primary care providers register a high rate of misdiagnoses and missed diagnoses. Howell et al. reported that the time from the appearance of first symptoms to the first instance of seeking medical help ranged from 1 to 7 months, and the time from help-seeking to diagnosis ranged from 2 weeks to 17 months in MM patients. Patients reported between one and ten primary care consultations with what they considered (in hindsight) to be myeloma symptoms, before the referral leading to diagnosis (3). The delay in diagnosis will deprive the patient of the optimal opportunity for treatment and can lead to the development of complications which can only at times be reversed. Increased tumor burden, symptoms, and organ damage all affect the treatment results and the capacity for myeloma patients to receive treatment (4). Improving the time to diagnosis of MM is a sine qua non condition to fulfill to give patients a fair chance of recovery especially in community hospitals and primary care clinics.

Being human-made, artificial intelligence (AI) can simulate intellectual work such as humans’ thoughts and judgments and has thus revolutionized the medical field (5). Hence, there is an increasing attention on the application of AI for the diagnosis and treatment of cancer (6, 7). In terms of settling the problems of classification and regression, the gradient boosting decision tree (GBDT) is regarded as a powerful ensemble learning technique (8). This model outperforms other models as the direction of the negative gradient is followed in order to train the residuals of each iteration, which can avoid the over-fitting problem. Furthermore, the GBDT model has performed well for knowledge discovery in various fields (9, 10). The current study is the first time that the artificial intelligence technology, including GBDT, has been used constructing a multiple myeloma early screening model based on a large amount of clinical conventional examination data. With the help of AI technology, the knowledge and experience of authoritative experts will better benefit the public, and effectively improve the current diagnosis rate of myeloma in the areas short of experience, which is of very important clinical significance.

## Patients and Methods

The Medical Ethics Committee at the Shengjing Hospital of China Medical University approved the present study (2020PS055J) according to the principles of the Declaration of Helsinki. The requirement for personal informed consent has been waived by the Ethics Committee as electronic medical records are researched retrospectively.

### Patient and Data Selection

In this retrospective research, our institutional databases were screened to investigate the patients admitted to our hospital for the first routine blood checks, hepatic function panel, renal function tests and immunoglobulin tests from January 2010 to January 2020. These included 1,741 records of multiple myeloma (MM) and 2,446 records of non-myeloma (infectious disease, rheumatic immune system disease, hepatic disease and renal disease). We also collected the data for these laboratory items from January 2020 to November 2020, including 68 records of newly diagnosed multiple myeloma (MM) and 70 records of non-myeloma aimed at testing the theory of generalizability. The diagnosis was made based on the 2014 International MM Working Group criteria (IMWG) (11). Nine variables (hemoglobin, serum creatinine, serum calcium, immunoglobulin (A, G and M), albumin, total protein, and ratio of albumin to globulin) were collected based on the use of current diagnostic criteria and medical judgment. Because immunoglobulin assays are not part of routine laboratory tests, we have therefore used six variables to try the alternative, cheaper model.

### Data Processing

Based on the diagnostic criteria and doctor-assisted judgments, the related factors for Multiple Myeloma risk prediction have been determined, and the original data related to the prediction have been extracted from the HIS and LIS databases. After extracting the correlating factors, the original sample set could not be directly applied in training machine learning models as the sample set still required further preprocessing of data.

### Handling the Missing Value in the Sample Set

The presence of empty values in the extracted raw data is first confirmed. A patient is eliminated from the pool when the number of missing values is larger than the designated threshold. We initially tested the number of missing values from 0–8. The results are depicted in **Supplementary Figure 1**. It was concluded that with the increase of the threshold value, F1 score increases followed by a decrease. This is due to the fact that fewer samples remain after data cleaning through the reduction of the number of the threshold, which results in the weakening of the generalization ability. However, when setting the threshold value as a sufficiently large value, more lost features are filled with normal values, resulting in the decrease of the F1 score. Considering these two factors, we finally set the threshold as 3 in our algorithm. If more than three factors are empty, the sample will be deleted, and if the missing value is below or equal to 3, this sample is deemed as fit to be retained. Through application of the same method, when six variables are considered, the threshold value is set to 2. Due to the fact that reserved data still contain missing values, its missing item will be filled with a normal value. With this missing data processing, we thus can reduce the possible deviation caused by using an abnormal value, and ensure there are no missing values in this data either for training or testing. We have implemented this method as a specific data processing module in our designed learning model.

### Expanding the Number of Positive Classes

**Figure 1:**
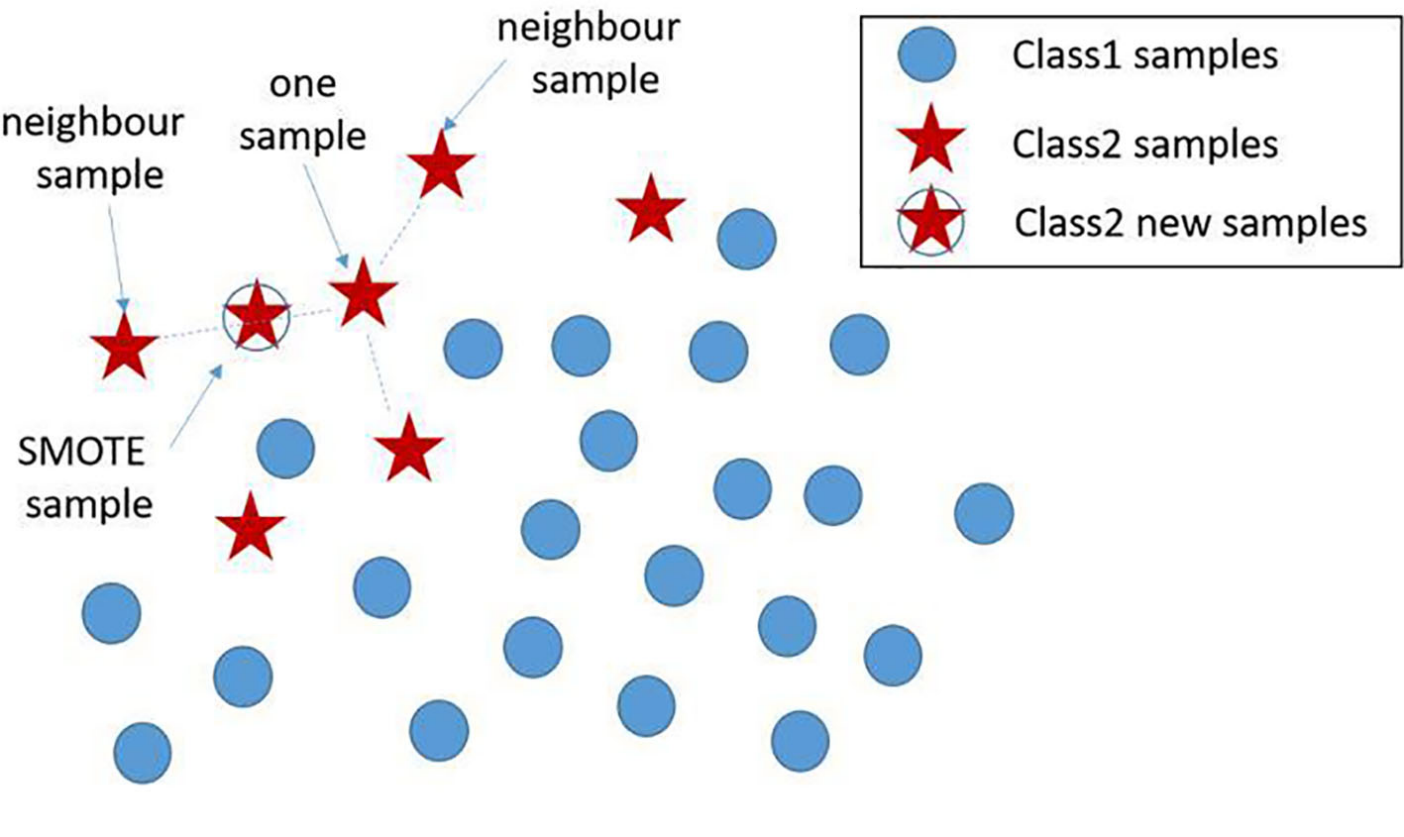
New sample generation with SMOTE algorithm. A new sample was generated using two existing samples, where the newly generated samples are denoted by ‘stars’. SMOTE, Synthetic Minority Oversampling Technique.

Based on the real data extracted from the system, the number of positives is much lower than the negatives. The Synthetic Minority Oversampling Technique (SMOTE) algorithm can be used to fix this unbalanced classification problem, by producing synthetic examples to increase the number of positive classes (12). The SMOTE algorithm analyzes and simulates the minority samples, uses the k-nearest neighbor (KNN) algorithm to synthesize new minority samples and adds the synthesized new samples into the training data, which can expand the sample size (13). The following steps are carried out by the SMOTE algorithm to synthesize new samples: the nearest neighbor algorithm is used and the number of the nearest neighbors for each minority is calculated; random number of samples are selected to randomly implement linear interpolation, and construct new minority samples; finally, new samples are synthesized with original data to produce new training sets (**Figure 1**). Here we obtained 580 synthetic samples based on 1,741 myeloma samples with SMOTE algorithm. These 580 synthetic samples were further integrated with original positive samples for model construction and testing.

### Expanding the Number of Correlation Features

Since the newly generated features can reflect the deviation degree of the detection items to its normal range, we thus utilize this feature as the part of the features to build the model. It should be noted that each index feature has a normal range in our system. Subsequently, the data standardization method was expanded by subtracting the upper and lower limits with the normal value. Moreover, we applied the outlier detection method after standardization method to reduce some outlier values in the sample data set. The relationship between the testing instance and its normal reference range is used to carry out feature correlation and expansion. For example, we assume the detection value of this feature index i as *d_i_*. Each index feature has a normal range $\left[L_{d}^{i},U_{d}^{i}\right]$. By comparing the differences between *d_i_* and $L_{d}^{i}$ and between $U_{d}^{i}$, two novel features $l_{d}^{i}$ *and* $u_{d}^{i}$ are generated by

(1) $$l_{d}^{i}=\{0,{\text{ d}}_{i}>L_{d}^{i}d_{i}-l_{d}^{i},\text{ otherwise}$$

(2) $$u_{d}^{i}=\{0,{\text{ d}}_{i}{\text{>L}}_{d}^{i}\;U_{d}^{i}-d_{i},\text{ otherwise}$$

where $l_{d}^{i}$ and $u_{d}^{i}$ reflect the deviation of the detection value from the normal range. The greater the detection value from the normal range, the larger $l_{d}^{i}$ or $u_{d}^{i}$ will be obtained. By utilizing the $l_{d}^{i},u_{d}^{i},l_{d}^{i}$ as the input features, more detailed and expanded features are thus obtained. Experiments show that this method can reduce the prediction variance of the model.

### Building the Prediction Model

Ensemble learning is a popular paradigm employed to leverage the strength of individual classifiers and mitigate their weaknesses. Ensemble techniques consist of combining more than one single classifier under a specific combination rule to solve the same task (14). As a common algorithm for ensemble learning, GBDT is composed of Decision Tree and Gradient Boosting (15) Because this tree model is characterized by high bias, low variance and small depth, highly pruned version of CART tress is thus utilized as the base classifiers for GBDT in each iteration (16).

The aggregated classifier using the additive modeling structure is as follows (17):

(3) $${\hat{y}}_{i}=F(x_{i})=\Sigma_{k=1}^{K}\gamma_{k}f_{k}(x_{i})$$

where ${\hat{y}}_{i}$ and *F*(*x_i_*) represent the predicted value of ith sample, *γ_k_* represents the weight of the kth tree, *f_x_*(*x_i_*) represents the prediction result of the kth regression tree for samples, *x_i_* represents the independent values used in fitting each regression tree, K is the number of CART model trees.

For Binary Classification problems, logarithmic loss function, which is also called as the log-likelihood loss function is utilized as the loss function:

(4) L(y,F(x))=log(1+exp(−2yF(x)))

where *F*(*x*) is given by Equation (3), and *x* is a generalization of *x_i_*, *y* represents the true value of the sample

With this loss function, the common applied gradient descent method is applied to find the optimal model. By calculating the negative gradient, we can obtain the moving direction brings has the steepest decline in the value of the loss function. The optimal model, through an iterative manner, can be found in this moving direction. With each iteration, the gradient descent method first calculates the negative gradient of the current model on all samples, and then trains a new base classifier with the value as the target for quasi merging, thus to calculate the weight of the base classifier. By utilizing this method iteratively, we finally realize the updating of the model.

**Figure 2:**
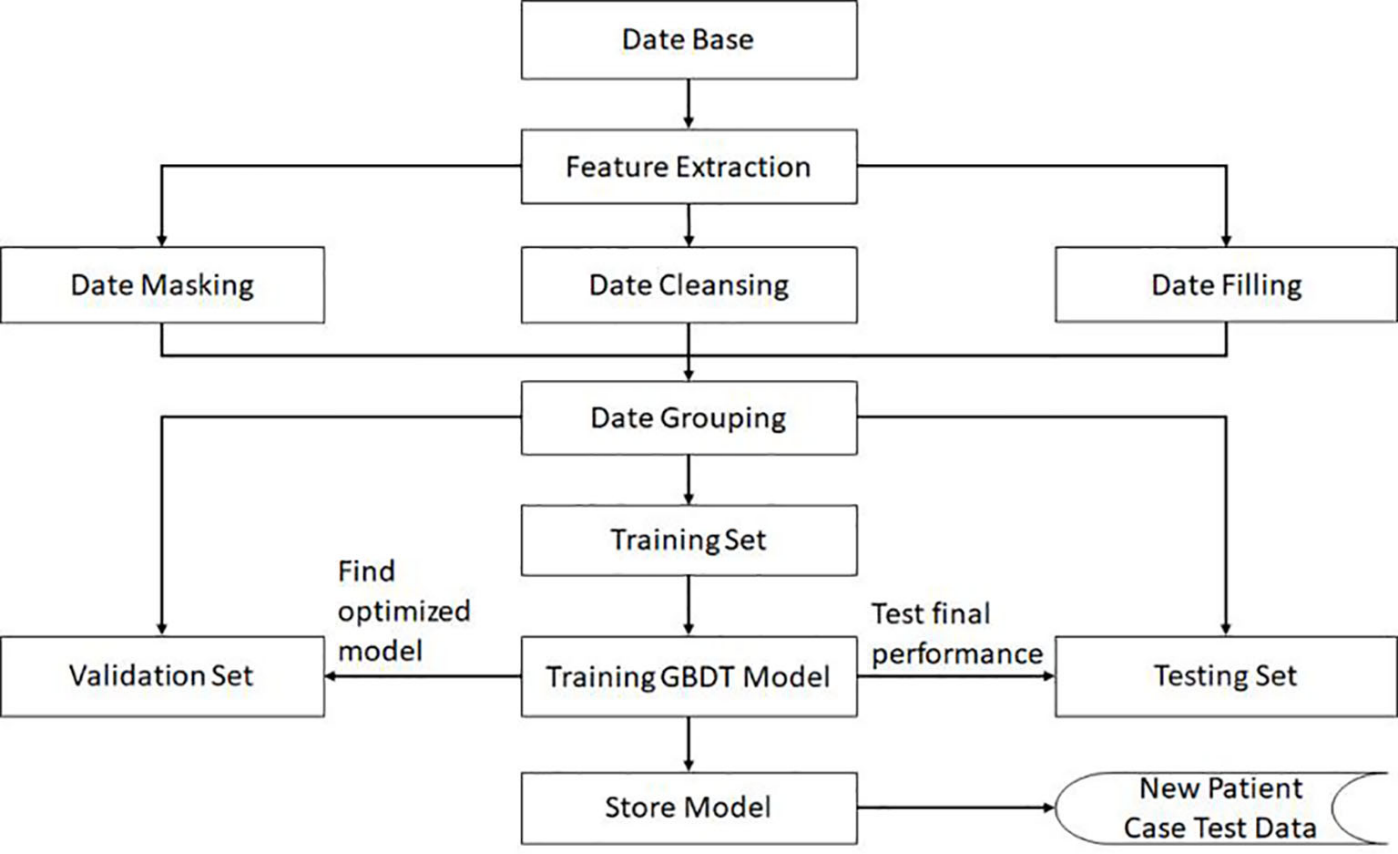
The flowchart and the complete training pipeline of the GBDT model.

In order to ensure the generalization ability of the model, the negative data and positive data are first mixed and shuffled, thus changing the original order. Then, using random extraction we obtain the training set and test set, which can ensure the independence of these two data sets. In our algorithm, the data volume ratio of these two data sets is 4:1. For the GBDT algorithm, the important super parameters include the maximum depth of the decision tree and the number of decision trees. The grid search method is then applied on the validation set, and the calculated optimal number of decision trees is 81, while the maximum depth for the decision trees is 6. All these performance results are obtained in the test data set. The complete training pipeline is demonstrated in **Figure 2**.

Moreover, Support Vector Machine (SVM) (13), Deep Neural Networks (DNN) (18), and Random forest (RF) (19) were also applied for performance comparisons. These three algorithms used the same training set and test set. For SVM algorithm, the Gaussian kernel function was utilized and the “gamma” parameter was set to 1. Gamma is a regularized super parameter. A larger gamma value denotes a more irregular decision boundary. Concurrently, a smaller gamma denotes a smoother boundary. Consequentially, we found that the gamma value needs to be adjusted when the model is over-fitted or otherwise (13). For DNN, we found that the model with more than four hidden layers seemed to be over fitted, and two hidden layers would not fit well. Therefore, a network with three hidden layers was constructed, where each layer contained 256 neurons and the ReLU activation function was applied. For random forest algorithm, we had tried the number of trees within set {50,100,300,500,600,800,1,000} and the depth of trees within set {5,10,15,20,30,50}. By testing all the combination on the validation set, we set the number of trees as 500 with its depth as 15.

Precision P, recall R, and F1 score are three common metrics used to evaluate the performance of a model in Machine Learning. Their formulas are as follows:

(5) $$P=\frac{TP}{TP+FP}$$

(6) $$R=\frac{TP}{TP+FN}$$

(7) $$F_{1}=2\times \frac{P\times R}{P+R}$$

From the formula, P stands for the proportion between the number of correctively predicted positives myeloma and the total number of positives in both myeloma and non-myeloma classes, P therefore represents the prediction of myeloma in our model; R stands for the proportion between the correctively predicted myeloma and the total number of actual myeloma patients; and F1-score is the harmonic mean of precision and recall (20). High F1-score only occurs when both recall and precision are high.

Among them, the clinical interpretation for evaluation criteria is as followed: For the category of myeloma, true positives (TP) indicate that the myeloma patient is correctly predicted to be in this myeloma class, a false positive (FP) indicates that it is not a myeloma patient and it has been incorrectly predicted as a myeloma class, and false negatives (FN) is the total number of incorrect predictions for a certain true myeloma class. For the non-myeloma category, TP represents the non-myeloma is correctly predicted in the non-myeloma class, FP represents the number of samples predicted as non-myeloma but actually myeloma, FN represents the number of non-myeloma incorrect predicted to be myeloma. The threshold for calculating TP and FN is the default value of 0.5.

The balance between the positive and negative samples can potentially cause the model to be impartial to positive and negative cases. Conversely, if the number of negative samples is larger than the number of positive samples, it will result in the deviation to the negative direction due to the over-exposure to negative samples. In our algorithm, the enhanced data is treated as normal data for model construction and testing without special treatment.

The curve of the receiver operator characteristics (ROC) is another important evaluation metric with regard with binary classification problems, and is a probability curve that plots the true positive rate (TPR) against false positive rate (FPR) at various threshold values.

All the experimental programs in this paper have been written in the Python language, and Python version 3.6 was applied as the interpreter. The machine learning development kit using in this paper is scikit learn version 0.20. The random forest applied in this paper is based on sklearn. ensemble class. The support vector machine is based on the SVM class of sklearn, and DBDT is based on Gradient Boosting Classifier class of sklearn. ensemble. The deep learning is operated on the tensorflow 1.12, with numpy 1.15.4 used to process and transform arrays, and Matplotlib 3.0.2 used to draw ROC curves.

## Results

Some 1,741 records of multiple myeloma and 2,446 records of non-myeloma (infectious diseases, rheumatic immune system diseases, hepatic disease and renal disease) were analyzed. The basic assay indicators are shown in **Table 1**.

**Table 1:** Subject characteristics.

Variable	Multiple myeloma dataset	Control dataset
	Mean (SD)	Mean (SD)
Creatinine (umol/L)	137.97 (4.62)	119.85 (2.83)
Serum β_2_ microglobulin (mg/L)	7.51 (0.30)	6.12 (0.66)
Urine β_2_ microglobulin (mg/L)	22.35 (1.24)	16.75 (5.16)
IgA (g/L)	4.43 (0.37)	2.89 (0.04)
IgG (g/L)	14.45 (0.48)	12.11 (0.14)
IgM (g/L)	0.67 (0.80)	1.23 (0.02)
Albumin (g/L)	35.91 (0.26)	29.70 (0.30)
Total protein (g/L)	68.60 (0.63)	58.93 (0.46)
Serum calcium (mmol/L)	2.20 (0.08)	2.04 (0.00)
Hemoglobin (g/L)	107.38 (0.70)	114.59 (0.47)

Moreover, we compared performance with or without data standardization and outlier detection. The results are shown in **Supplementary Table 1**. The contents of the table indicate performance comparison of utilizing three features $u_{d}^{i}$, *d_i_* and $l_{d}^{i}$ or utilizing only feature *d_i_* with multiple tests, where both the mean value and standard deviation are compared. As can be seen from the statistics, using $u_{d}^{i}$, *d_i_* and $l_{d}^{i}$ can reduce the standard deviation. It can be concluded that our method increases the reliability of prediction performance. By taking the F1 scores of positive samples as example, a smaller standard deviation is yielded, indicating the estimation is more stable.

For comparison, SVM, DNN, RF and GBDT models were trained and tested on the same dataset using nine variables as hemoglobin, serum creatinine, serum calcium, immunoglobulin (A, G and M), albumin, total protein, and ratio of albumin to globulin. Among the four machine learning algorithms, GBDT yielded the highest precision 0.929 and 0.899 for the myeloma and non-myeloma respectively. GBDT also has the highest recall (0.900) and F1 score (0.915) for myeloma. The value of P, R, and F1 of the four machine learning algorithms are shown in **Table 2**, and can be calculated according to (21). The influence weight of each variable on classification calculated by GBDT is shown in **Supplementary Table 2**. The weight of each feature in the table is automatically calculated by machine learning algorithm, which shows the importance of each feature for disease prediction. A larger value indicates a bigger influence on classification by this variable.

**Table 2:** Results of Testing Group based on 9 variables.

Method	Class	P	R	F_1_
GBDT	Non-myeloma	0.899	0.928	0.913
	Myeloma	0.929	0.900	0.915
RF	Non-myeloma	0.884	0.903	0.908
	Myeloma	0.901	0.90	0.906
SVM	Non-myeloma	0.830	0.827	0.829
	Myeloma	0.836	0.839	0.837
DNN	Non-myeloma	0.850	0.783	0.815
	Myeloma	0.772	0.842	0.805

An immunoglobulin assay is not part of routine laboratory queries, we have therefore used six variables namely: hemoglobin, serum creatinine, serum calcium, albumin, total protein, and ratio of albumin to globulin) to train the model. If the immunoglobulin were considered unwarranted, the model would eventually have 0.797 precision, 0.726 recall and 0.760 F1 score, which are lower than the nine variable model fit with immunoglobulin based on the GBDT model. The value of P, R, and F1 related to six variable data were shown in **Supplementary Table 3** and **Supplementary Figure 2**. For the clinics and hospitals where immunoglobulin (A, G and M) can be routinely measured, the 9-variable model should be used to achieve higher accuracy. For health centers where immunoglobulin examination is not commonly ordered, the 6-variable model should be applied as a precautionary measure should indications of further immunoglobulin investigations appear.

**Figure 3:**
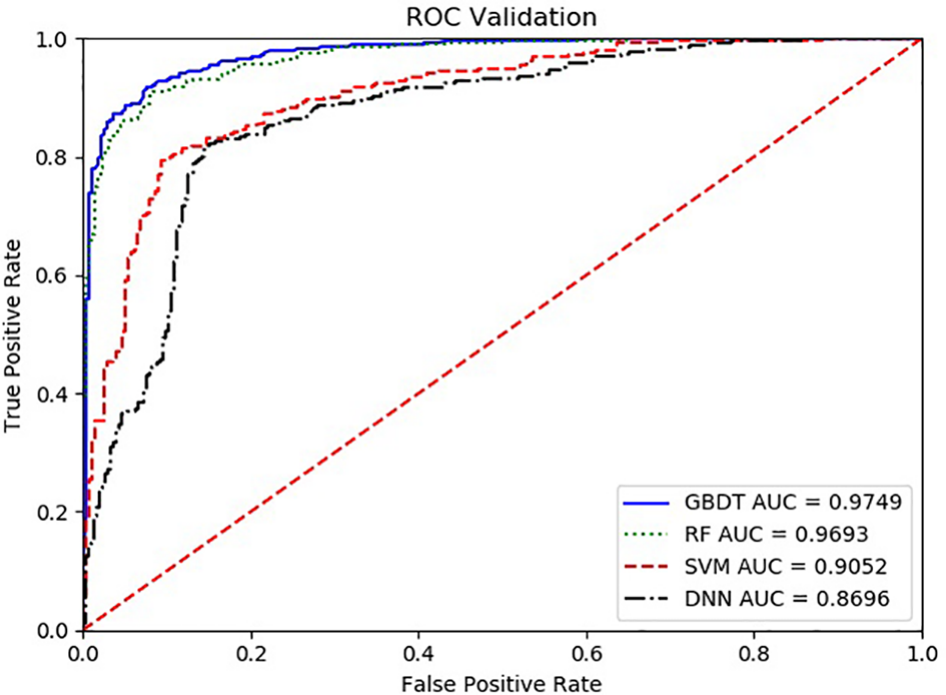
The ROC comparison of four algorithms based on nine variables. The classifier with GBDT obtains an AUC of 0.975 [95% confidence interval (CI): 0.986–0.963], and has the best performance when comparing with the other three algorithms. ROC, Receiver Operating Characteristic; GBDT, Gradient Boosting Decision Tree; RF, Random Forest; DNN, Deep Neural Networks. Nine items are hemoglobin, serum creatinine, serum calcium, immunoglobulin (A, G and M), albumin, total protein, and ratio of albumin to globulin.

With the ROC curve, the area under the curve (AUC) can be calculated to measure a classifier’s ability to distinguish classes. The higher the AUC, the better the model will be at classifying. **Figure 3** illustrates the performance of comparing the AUC with these four algorithms. We can observe that the classifier with GBDT obtains an AUC of 0.975 [95% confidence interval (CI): 0.963–0.986], and has the best performance when compared to the other three algorithms.

The model we trained was also tested on the new data set, and the performance index obtained is similar with the result on the original data set, but for January to November in 2020. GBDT once more showed the highest recall (0.909), precision (0.952) and F1 score (0.930) for the myeloma set. Also, GBDT showed recall (0.954), precision (0.912) and F1 score (0.932) for the non-myeloma set with the threshold 0.5. The values of P, R, F1 of the 68 newly diagnosed MM and 70 non-myelomas by machine learning algorithms are shown in **Supplementary Table 4**. We can observe that the classifier with GBDT obtained an AUC of 0.974 in the new data shown in **Supplementary Figure 3**. From this analysis, it can be inferred that the model trained by the original data set has a strong generalization ability.

## Discussion

Multiple myeloma has an incidence rate of about 28,000 new cases per year in China (22) and due to various clinical presentations, diagnosis is a challenge. Data shows that more than 90% of patients suffer from bone pain and fractures in the early stages of the disease or during the course of disease progression, while about 50% of patients have renal impairment (23–25). The public do not understand MM and with a slow onset of the disease and a lack of typical symptoms in China, MM can hardly be distinguished from other diseases in other departments. This thus leads to delayed treatment and the poor prognosis of patients (26). In order to improve early diagnosis of MM, IMWG recommends the application of a percentage of clonal plasma cell into the bone marrow, serum-free light chain ratios and MRI focal lesions as additional biomarkers for the disease (27). However, in China, these programs are not considered routine inspection programs, and some primary medical care centers do not even carry out the relevant tests. We hope that based on the integration of data from routine laboratory tests in clinics, an early warning system for MM can be designed to make it easier to proceed.

As artificial intelligence develops at an extraordinarily pace, countless applications have been created in the past decade (28–30). Recently, AI has been increasingly adopted to diagnose and predict some diseases, while the medical image analysis community has paid particular attention to the success of machine learning in computer vision (31, 32). Some researchers have been initiated into applying AI techniques to the quantification of early rheumatoid arthritis using Magnetic Resonance Imaging (MRI) data (33). Ni et al. achieved a general accuracy of 84.48% when using radionics analysis based on the LASSO + GBDT method for the noninvasive diagnosis of microvascular invasion in hepatocellular carcinoma [34]. Zhang et al. used LASSO + GBDT to examine the ability of radionics characteristics from MRI in differentiating anaplastic oligodendroglioma (AO) from atypical low-grade oligodendroglioma (35). The majority of researchers have performed quantitative analysis of multi-modality image data for diagnosis and prognosis by using artificial intelligence methods, but few of them have focused on the routine laboratory tests that easily obtained from clinic.

AI techniques have been applied to the treatment of multiple myeloma. Ji et al. constructed a hybrid multi-scale agent-based model (HABM) model to provide new insight into the development of myeloma in a bone marrow micro-environment that is the basis of the immune system, and also build an efficient computational platform for prediction of drug response for discovering the optimal dose combination (36). Zhang et al. built a more efficient approach by combining the standard ordinal logistic regression and the hierarchical modeling. This method can simultaneously analyze numerous variables for detecting important predictors and for predicting multi-level drug response (37). Tang et al. established and validated a novel mathematical model of multiple myeloma cell dynamics. The clinical data compounded with mathematical modeling, suggested that bortezomib-based therapy exerted a selection pressure on myeloma cells (38) Bouchnita et al. developed a hybrid discrete-continuous model to predict the response of MM tumors to treatment with gefitinib and 6-aminonicotinamide (6-AN) (39). There is no published early diagnosing, laboratory results-based AI models as of yet.

According to our preliminary test based on the data of 1,000 myeloma patients and 2,000 non-myeloma patients in our hospital, the predictive value of artificial intelligence can reach more than 90% with the prospect of having a wide application. Our research also indicates that the SVM algorithm is suitable for classifying small-size data, while the DNN algorithm is suitable for classifying large-size data. By efficiently extracting the sample features, the GBDT algorithm can simultaneously train some decision trees on the ability to sort out the features based on their importance, so as to obtain the best performance when comparing with the other three algorithms (40).

Taking the integration of test data as the breakthrough point, this project adopts the methods of big data analysis and artificial intelligence, so as to propose the automatic integration of routine test reports, establish multiple myeloma screening models, give early warnings for multiple myeloma, and improve the diagnosis rate. The research contents are innovative in the medical, information and business fields.

## Conclusion

In this study, routine exam results obtained from general hospitals are utilized to train machines to realize automatic screening, identify patients at a high risk of diagnosed multiple myeloma and provide early warnings through the big data platform, artificial intelligence and other technologies. This technology can be widely used in general hospitals and primary medical care to improve the early diagnosis rate of myeloma and prevent the occurrence of missed diagnosis and misdiagnosis. At the end, an early warning and screening system for myeloma based on artificial intelligence will be formed.

## Data Availability Statement

The original contributions presented in the study are included in the article/**Supplementary Material**. Further inquiries can be directed to the corresponding author.

## Ethics Statement 

Written informed consent was obtained from the individual(s) for the publication of any potentially identifiable images or data included in this article.

## Author Contributions

Study conception and design: WYan and HW. Literature review and data extraction: HS. Quality control: WYan and AL. Model construction and statistical analysis: JC and TH. Manuscript preparation: WYan and HW. Manuscript review: WYan, AL, WYang, and HW. Editing and revisions: WYan, HW, JC, TH, and CW. All authors contributed to the article and approved the submitted version.

## Funding 

This work was supported in part by the National Natural Science Foundation of China under Grants 61972079 and 61772126, in part by the Shingling LiaoNing Revitalization Talents Program under Grant XLYC1802100, and in part by the Key R&D program of Liaoning Province under Grant 2019JH2/10100027.

## Conflict of Interest

The authors declare that the research was conducted in the absence of any commercial or financial relationships that could be construed as a potential conflict of interest.

## Supplementary Material

Supplementary Figure 1 Performance comparison with different threshold in the four algorithms.

Supplementary Figure 2 The ROC comparison of four algorithms based on 6 variables. Six variables are hemoglobin, serum creatinine, serum calcium, albumin, total protein, and ratio of albumin to globulin.

Supplementary Figure 3 The ROC comparison of four algorithms based on 9 variables from new cases set in 2020.
