# Supplementary figures and images for: Employment of Artificial Intelligence Based on Routine Laboratory Results for the Early Diagnosis of Multiple Myeloma
Source: Front Oncol. 2021 Mar 29;11:608191. doi: 10.3389/fonc.2021.608191 (PMC8039367; doi:10.3389/fonc.2021.608191)

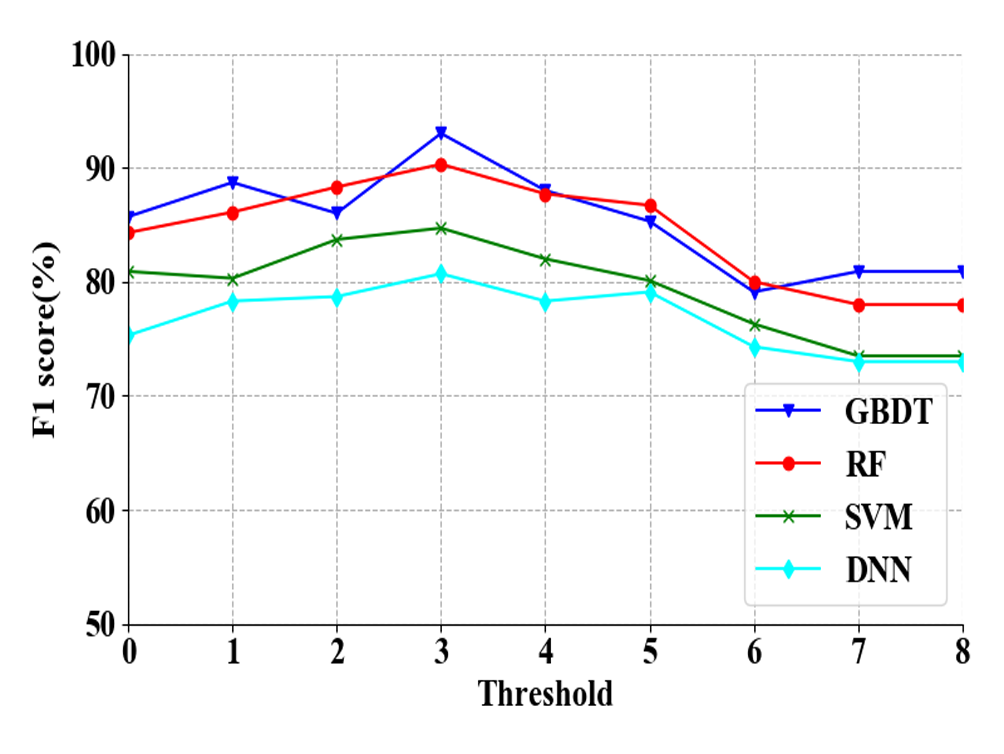

Supplement: Supplementary Figure 1 — Performance comparison with different threshold in the four algorithms. [file Image_1.tif]

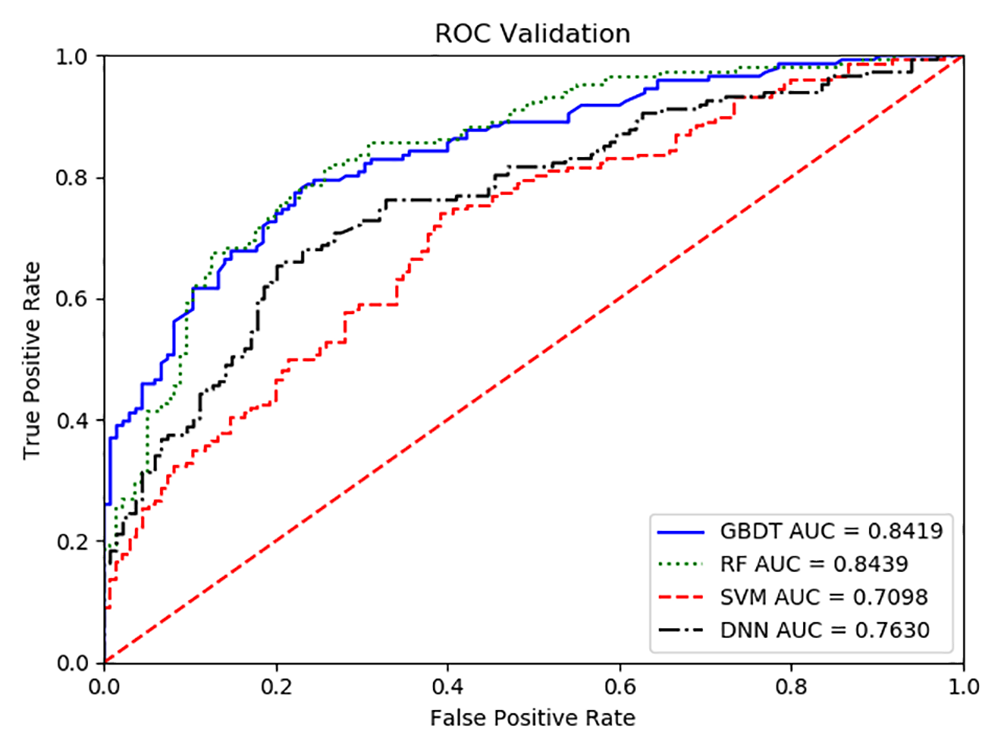

Supplement: Supplementary Figure 2 — The ROC comparison of four algorithms based on 6 variables. Six variables are hemoglobin, serum creatinine, serum calcium, albumin, total protein, and ratio of albumin to globulin. [file Image_2.tif]

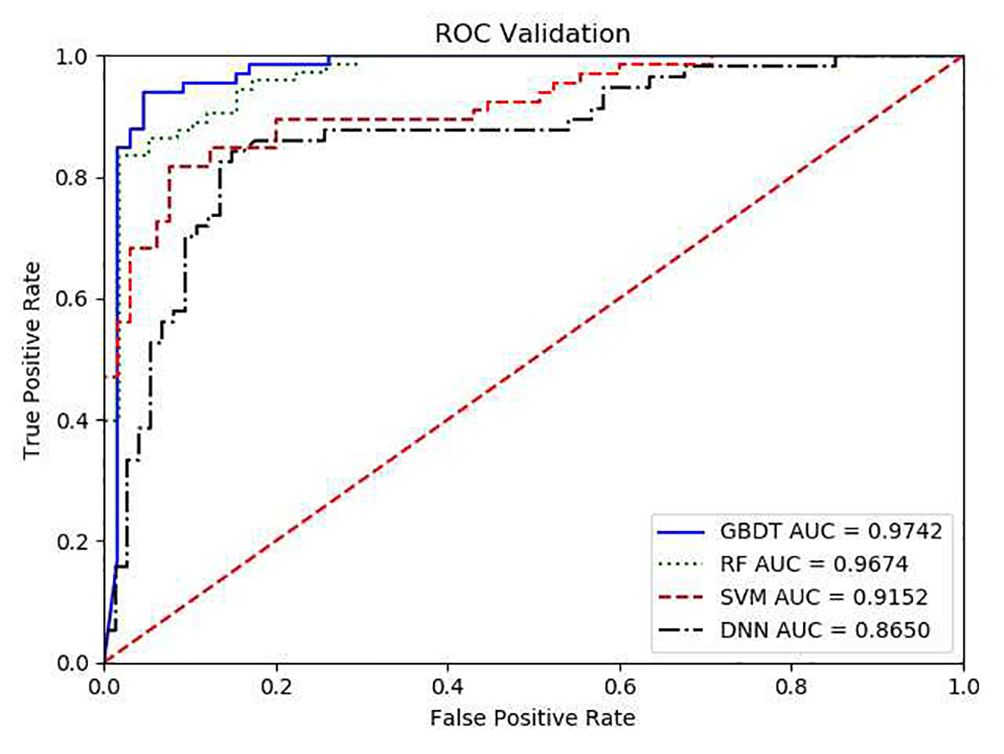

Supplement: Supplementary Figure 3 — The ROC comparison of four algorithms based on 9 variables from new cases set in 2020. [file Image_3.tif]
