# Supplementary material for: Employment of Artificial Intelligence Based on Routine Laboratory Results for the Early Diagnosis of Multiple Myeloma
Source: Front Oncol. 2021 Mar 29;11:608191. doi: 10.3389/fonc.2021.608191 (PMC8039367; doi:10.3389/fonc.2021.608191)
Supplement: Supplementary file 4 [file Table_1.docx]

**Supplement Table 1. Performance Comparison with or without Data Standardization and Outlier Detection.**

|  | **With data standardization and outlier detection** | | **Without data standardization and outlier detection** | |
| --- | --- | --- | --- | --- |
|  | Mean value | Standard deviation | Mean value | Standard deviation |
| GBDT | 0.928 | 0.014 | 0.918 | 0.055 |
| RF | 0.897 | 0.012 | 0.901 | 0.036 |
| SVM | 0.838 | 0.022 | 0.839 | 0.046 |
