# Supplementary material for: Employment of Artificial Intelligence Based on Routine Laboratory Results for the Early Diagnosis of Multiple Myeloma
Source: Front Oncol. 2021 Mar 29;11:608191. doi: 10.3389/fonc.2021.608191 (PMC8039367; doi:10.3389/fonc.2021.608191)
Supplement: Supplementary file 5 [file Table_2.docx]

**Supplement Table 2.** **The influence weight of each variable on classification calculated by GBDT.**

| **Variables** | **The influence weight** |
| --- | --- |
| Hemoglobin | 0.038 |
| Serum creatinine | 0.045 |
| Serum calcium | 0.065 |
| Immunoglobulin A | 0.341 |
| Immunoglobulin G | 0.063 |
| Immunoglobulin M | 0.335 |
| Albumin | 0.028 |
| Total protein | 0.053 |
| The ratio of albumin to globulin | 0.032 |
