# Supplementary material for: Employment of Artificial Intelligence Based on Routine Laboratory Results for the Early Diagnosis of Multiple Myeloma
Source: Front Oncol. 2021 Mar 29;11:608191. doi: 10.3389/fonc.2021.608191 (PMC8039367; doi:10.3389/fonc.2021.608191)
Supplement: Supplementary file 6 [file Table_3.docx]

**Supplementary Table 3. Results of Testing Group based on 6 variables.**

| **Method** | **Class** | **P** | **R** | **F_1_** |
| --- | --- | --- | --- | --- |
| GBDT | Non-myeloma | 0.730 | 0.800 | 0.763 |
|  | Myeloma | 0.797 | 0.726 | 0.760 |
| RF | Non-myeloma | 0.757 | 0.779 | 0.768 |
|  | Myeloma | 0.787 | 0.766 | 0.776 |
| SVM | Myeloma | 0.630 | 0.644 | 0.637 |
|  | Myeloma | 0.664 | 0.651 | 0.657 |
| DNN | Non-myeloma | 0.696 | 0.702 | 0.699 |
|  | Myeloma | 0.726 | 0.721 | 0.724 |
