# Supplementary material for: Employment of Artificial Intelligence Based on Routine Laboratory Results for the Early Diagnosis of Multiple Myeloma
Source: Front Oncol. 2021 Mar 29;11:608191. doi: 10.3389/fonc.2021.608191 (PMC8039367; doi:10.3389/fonc.2021.608191)
Supplement: Supplementary file 7 [file Table_4.docx]

**Supplementary Table 4. Results of Testing Group on 9 variables from new cases set in 2020.**

| **Method** | **Class** | **P** | **R** | **F_1_** |
| --- | --- | --- | --- | --- |
| GBDT | Non-myeloma | 0.912 | 0.954 | 0.932 |
|  | Myeloma | 0.952 | 0.909 | 0.930 |
| RF | Non-myeloma | 0.891 | 0.845 | 0.867 |
|  | Myeloma | 0.882 | 0.918 | 0.899 |
| SVM | Myeloma | 0.826 | 0.877 | 0.851 |
|  | Myeloma | 0.871 | 0.818 | 0.844 |
| DNN | Non-myeloma | 0.842 | 0.865 | 0.853 |
|  | Myeloma | 0.818 | 0.790 | 0.804 |
